# Extended total gastrectomy after nivolumab for unresectable multivisceral invasive gastric cancer

**DOI:** 10.1186/s40792-020-01040-3

**Published:** 2020-11-25

**Authors:** Satoshi Toyota, Hiroshi Naito, Saki Motoyoshi, Ryota Nakanishi, Eiji Oki, Hiroyuki Orita, Daisuke Korenaga

**Affiliations:** 1 Department of Surgery, Nakatsu Municipal Hospital, Nakatsu, Oita Japan; 2 Department of Surgery and Science, Graduate School of Medical Sciences, Kyushu University, 3-1-1, Maidashi, Higashi-ku, Fukuoka, 812-8582 Japan

**Keywords:** Gastric cancer, Nivolumab, Conversion surgery

## Abstract

**Background:**

Chemotherapy has been considered the main treatment for stage IV gastric cancer (GC). However, advances in chemotherapy have provided new clinical approaches, permitting conversion surgery with the aim of R0 resection after resolving unresectability issues.

**Case presentation:**

A 70-year-old man with gastric cancer invading the pancreatic tail and spleen and with periaortic lymph-node enlargement was admitted to our hospital. After 24 courses of nivolumab as third-line chemotherapy, periaortic lymph-node enlargement was resolved, and conversion surgery was planned. Intraoperatively, we found no peritoneal metastasis, but the distal pancreas, splenic hilum, and transverse colon were adhered to the gastric body. Therefore, we performed D2 total gastrectomy with distal pancreatosplenectomy and partial transverse colectomy. The pathological diagnosis was type III moderately differentiated tubular adenocarcinoma (tub2) with signet ring cells, stage ypT1b (SM), ly0, and v0. The pathological proximal and distal tumor margins were negative. One lymph-node metastasis was observed (No. 4d; 1/23). Postoperatively, no recurrence was observed over 7 months, without adjuvant chemotherapy.

**Conclusions:**

Nivolumab may allow R0 resection in patients with unresectable gastric cancer. Conversion surgery should be considered even after third-line nivolumab treatment.

## Background

Chemotherapy has been considered the main treatment for stage IV gastric cancer (GC). However, advances in chemotherapy have provided new clinical approaches, permitting conversion surgery with the aim of achieving R0 resection after resolving unresectability issues. Conversion gastrectomy for GC is uncommon, because stage IV GC is a heterogenous condition involving distant hematological metastasis, distant lymph-node metastasis, and peritoneal dissemination. Moreover, almost all conversion surgeries for GC are performed in patients experiencing dramatic responses to first-line chemotherapy [1]. Yoshida et al. [2] noted that this is because tegafur, gimeracil, and oteracil potassium-based regimens are necessary for a good response. However, a rare case of conversion gastrectomy after third-line chemotherapy with nivolumab (Bristol-Myers Squibb, Princeton, NJ and Ono Pharmaceutical, Trenton, NJ), a human immunoglobulin G4 programmed cell death protein 1-blocking monoclonal antibody, has been reported [3, 4].

We report extended surgery and total gastrectomy with pancreatectomy, splenectomy, and colectomy for advanced GC after nivolumab as third-line chemotherapy.

## Case presentation

**Fig. 1:**
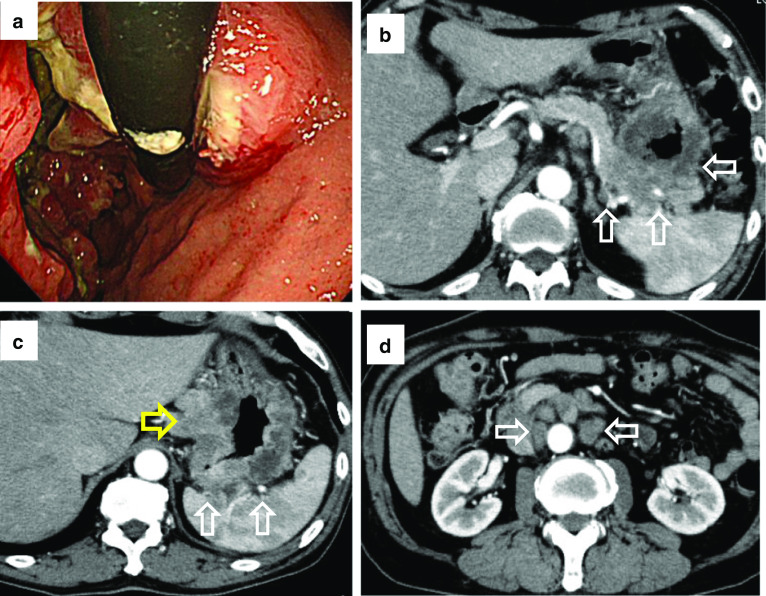
Endoscopic and computed tomography (CT) images before nivolumab therapy. **a** First gastric endoscopy examination showing Borrmann type 3 gastric cancer occupying the entire circumference of the gastric body. **b** First CT image showing the primary tumor invading the pancreatic tail (white arrows). **c** The spleen was also invaded (white arrows), and the lesser curvature lymph nodes were enlarged (yellow arrows). **d** Enlarged para-aortic lymph nodes (white arrows)

**Fig. 2:**
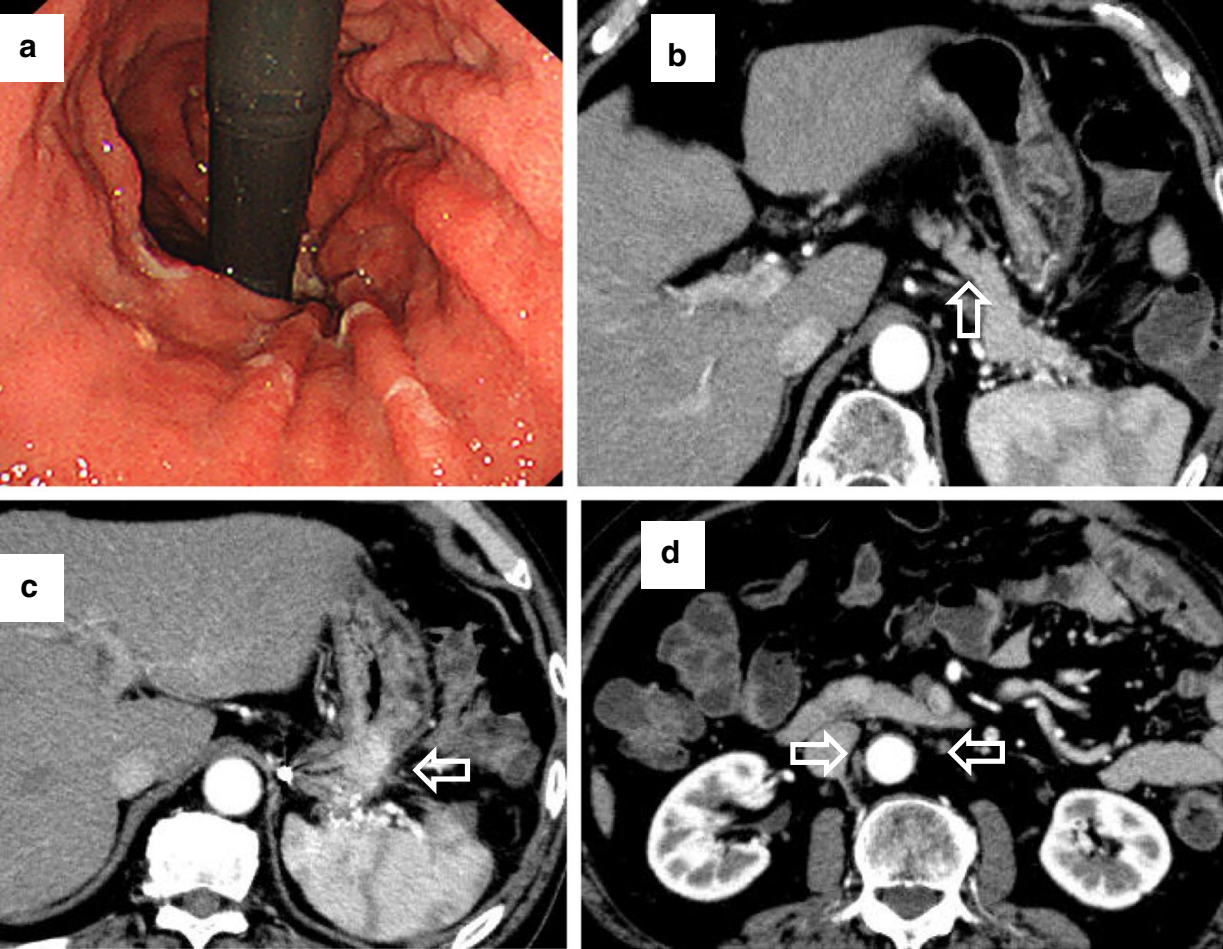
Endoscopic and computed tomography (CT) images after nivolumab therapy. **a** Endoscopic image showing that the primary lesion had decreased markedly in size. **b** CT image showing decreased invasion of the pancreas (white arrows). **c** CT image showing decreased invasion of the spleen, and smaller lesser curvature lymph nodes (white arrows). **d** CT image showing that the para-aortic lymph nodes are inconspicuous (white arrows)

A 70-year-old man was admitted to our hospital to undergo treatment for GC in October 2017. Preoperative endoscopy revealed a circumferential type 3 tumor extending from the upper to lower portions of the gastric body (Fig. 1a). Biopsy revealed moderately differentiated adenocarcinoma. Human epidermal growth factor receptor type 2 (HER2) immunohistochemical staining was negative. Preoperative computed tomography (CT) revealed a primary tumor invading the pancreatic tail and spleen and enlarged regional and para-aortic lymph nodes (Fig. 1b–d). The patient received tegafur, gimeracil, and oteracil potassium plus oxaliplatin as first-line chemotherapy. After three courses, CT revealed progression of the primary tumor. After seven courses of paclitaxel plus ramucirumab as second-line chemotherapy, the primary tumor had increased in size. Therefore, we initiated third-line chemotherapy with nivolumab at 240 mg/body. Before third-line nivolumab, the primary tumor was still invading the pancreatic tail, spleen, and transverse colon, and the regional and para-aortic lymph nodes had decreased in size, but had not returned to normal. Although immune-related adverse effects of a grade 1 rash and grade 2 adrenal gland hypofunction occurred after the fifth course, nivolumab was continued for 24 courses. After the 24 courses of nivolumab, endoscopy revealed that the primary tumor was smaller (Fig. 2a). Follow-up CT revealed that the regional and para-aortic lymph nodes had decreased in size by 73%, and the degree of invasion had improved (Fig. 2b–d); therefore, we planned conversion surgery. Intraoperatively, we found no intraperitoneal nodules indicating metastasis, and cytology revealed class II ascites. However, the distal pancreas, splenic hilum, and transverse colon were adhered to the gastric body, which was considered invasion by the primary tumor. Thus, we performed D2 total gastrectomy with distal pancreatosplenectomy and partial transverse colectomy. We retrieved 32 regional lymph nodes, but could not recover the para-aortic lymph nodes because of increased blood loss. The total operative time was 8 h and 50 min. Total blood loss was 1966 ml, and we administered transfusions of 560 ml erythrocytes and 480 ml fresh-frozen plasma (FFP).

The patient was transferred to the intensive care unit postoperatively. On postoperative day (POD) 5, after oral intake was started, he developed a fever and abdominal pain. CT revealed a pancreatic fistula and leakage from the transverse colon anastomosis; hence, we placed an endoscopic ultrasound-guided drain, and the fistula resolved on POD 79. Oral intake restarted on POD 82, and the patient was finally discharged from our hospital in good condition on POD 87.

**Fig. 3:**
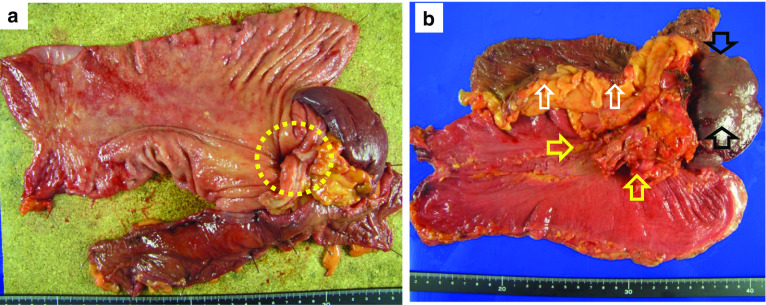
Resected specimen. **a** Mucosal side of the stomach. Only one section shows a small area of moderately differentiated adenocarcinoma (yellow circle). Stage: pT1b (SM), Ly0, and v0. **b** Serosal side of the stomach. Pancreas (yellow arrows), spleen (black arrows), and transverse colon (white arrows) are adhered to the gastric body

**Fig. 4:**
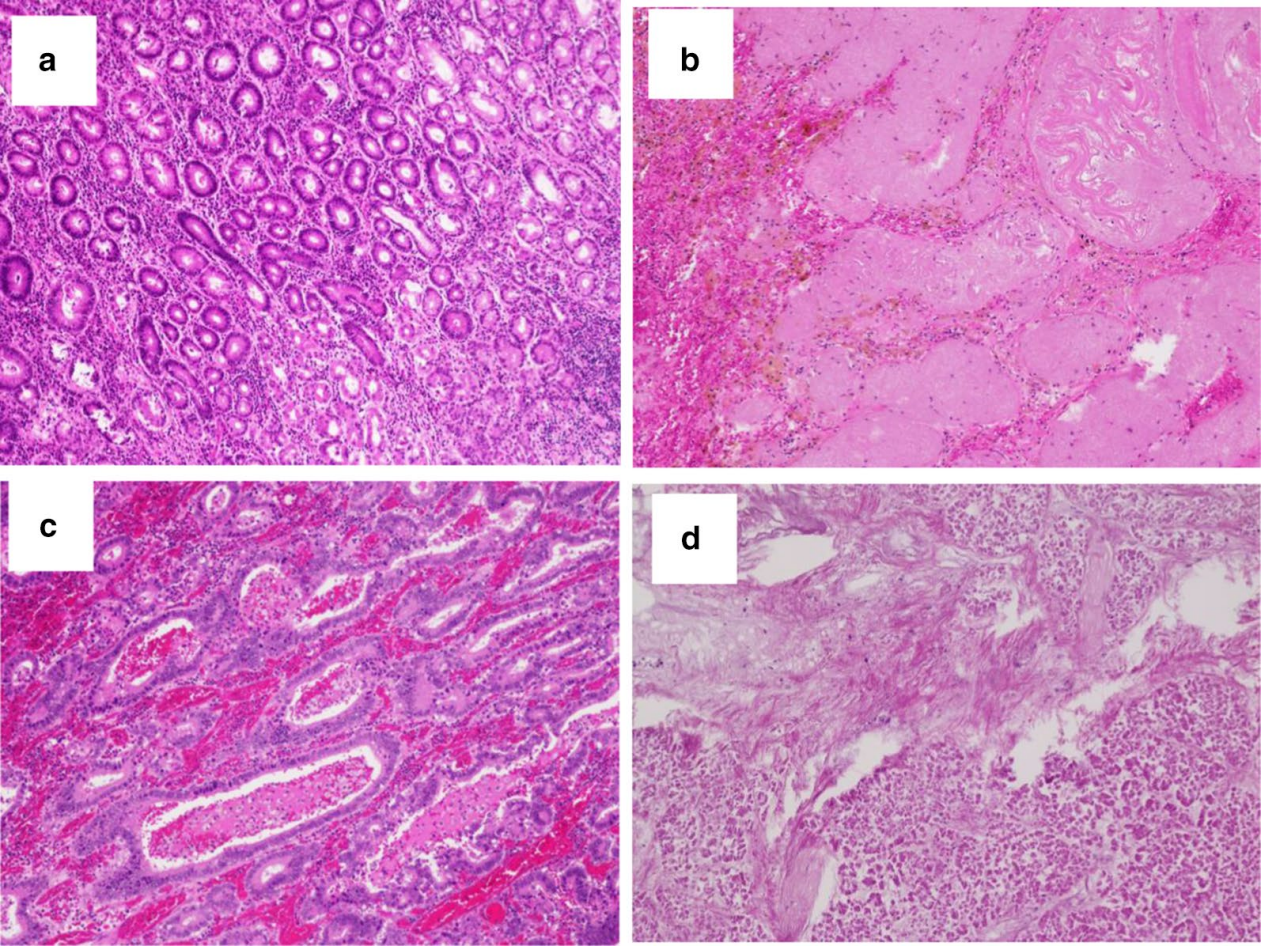
Photomicrographs of the hematoxylin and eosin-stained histological samples of the resected specimens from the gastric body, lymph node, and pancreas. **a** Only a small area in one section of the gastric body shows moderately differentiated adenocarcinoma (tub2). **b** Areas of fibrosis and necrotic foci are seen in other parts of the gastric body. **c** The No. 4d lymph node (1/3) showing carcinoma metastasis. **d** No carcinoma cells are visible in the pancreas, but necrosis and fibrosis are observed in large areas of the pancreas

**Fig. 5:**
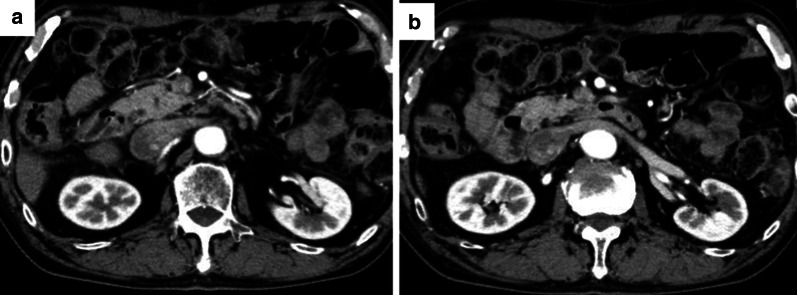
CT findings 7 months after surgery. **a**, **b** The para-aortic lymph nodes maintained a reduced state

The pathological diagnosis was type III, moderately differentiated tubular adenocarcinoma (tub2) with signet ring cells, stage ypT1b (SM), ly0, and v0. The pathological proximal and distal margins were free from carcinoma cells (Figs. 3a, b, 4a, b). One metastatic lymph node was observed (No. 4d node) (Fig. 4c). The therapeutic effect was Grade 2b, and there were no signs of recurrence on CT 7 months after surgery, without adjuvant chemotherapy (Fig. 5).

## Discussion

To our knowledge, this is the third report of conversion surgery for advanced GC after nivolumab as third-line chemotherapy [3, 4].

Patients with para-aortic lymph node and peritoneal metastases are considered unresectable, and the survival of these patients is poor [5]. However, some studies showed the efficacy of conversion surgery for advanced GC [6–8]. Fukuchi et al. [7] reported that the presence of just one noncurative factor and R0 conversion resection were significant independent predictors of good overall survival. Kinoshita et al. [8] reported that the 3-year overall survival rate of patients with a single incurable factor was significantly longer than for patients with multiple factors. Our patient had a single incurable factor, para-aortic lymph-node metastasis, and because of the efficacy of nivolumab, we judged that complete resection was possible; therefore, we performed conversion surgery. As for the timing of conversion surgery, we did not reach a consensus; however, Zurleni et al. [1] stated that conversion surgery should be performed to render unresectable factors controllable until chemotherapy resistance. Based on this, the timing of the conversion surgery was reasonable.

Previously, third-line chemotherapy was not expected to resolve unresectable factors for advanced GC patients and most conversion surgeries. However, we succeeded in this regard in two cases of conversion surgery after nivolumab as third-line chemotherapy, including the current case. The second case was a 75-year-old man with advanced gastric antrum cancer with peritoneal metastasis. Peritoneal metastasis was resolved after 23 courses of nivolumab, and he successfully underwent distal gastrectomy with D1+ dissection (R0 resection). No lymph-node or peritoneal metastasis was observed in resected specimens [3]; thus, nivolumab demonstrated high efficacy. Namikawa et al. [9] noted that progression-free survival in advanced GC patients with immune-related adverse events (irAEs) was significantly longer than that of patients without irAEs (5.8 months vs. 1.2 months, respectively; *P* = 0.028). Matsuda et al. [10] showed that the absence of irAEs (hazard ratio (HR) = 9.54, 95% confidence interval (CI) 3.34–27.30 for yes vs. no) was associated with a poor prognosis in GC patients. Our two cases experienced unique adverse events of Grade 1 chylous ascites in the first case and Grade 1 rash and Grade 2 adrenal dysfunction in the second case. It is difficult to diagnose irAEs; however, unique phenomena appearing during nivolumab therapy may be an indication of therapeutic effect.

Certainly, we must discuss the validity of extended resection. Intraoperatively, the primary tumor was thought to have invaded the pancreas, spleen, and transverse colon. However, only a small histological section showed carcinoma (pT1b (SM)) (Figs. 3a, 4a), and other parts that were considered invasion in the gastric body were actually necrosis and fibrosis (Fig. 4b, c). Additionally, no carcinoma cells were observed in the pancreas histologically, and necrosis and fibrosis occupied a large area (Fig. 4d). The therapeutic effect was Grade 2b; however, it is difficult to distinguish carcinoma invasion or adhesion intraoperatively. Furthermore, peeling and dissection to remove the tumor are not recommended to achieve R0 resection; therefore, we were unable to avoid combined resection of the distal pancreas, spleen, and transverse colon. As a result, grade IIIa pancreatic fistula and anastomotic leakage occurred postoperatively. Complications after curative surgery have a negative impact on the prognosis of gastric cancer patients [11]. Kinoshita et al. [8] reported that 11% of patients who underwent conversion gastrectomy after combined docetaxel and cisplatin, and tegafur, gimeracil, and oteracil potassium therapy developed grade IIIa complications after surgery. Aside from neoadjuvant chemotherapy, several studies demonstrated that the number of resected organs is associated with poor prognosis [12–14]. However, Fabio et al. stated that it is not the number of resected organs but the completeness of resection that is the strongest prognostic factor [15]. Similarly, the risk factors for extended surgery are not clear, but extended conversion surgery requiring two or more organ resections should be carefully considered.

No signs of recurrence were observed 7 months after surgery. A consensus has not been established for cases in which the para-aortic lymph nodes have re-swollen; however, we will re-administer nivolumab. Surgery for para-aortic lymph nodes is difficult because of adhesion after postoperative inflammation, and complications other than resistance to nivolumab were not observed.

## Conclusions

We presented a case of successful extended surgery for advanced GC and no recurrence 7 months postoperatively. Conversion surgery is a treatment option even after third-line nivolumab treatment; however, there is no consensus regarding the validity of conversion surgery for stage IV GC. Further follow-up and study are required.

## Data Availability

Not applicable.

## Abbreviations

IrAEs: Immune-related adverse events
GC: Gastric cancer
